# Quality of life after carotid endarterectomy

**DOI:** 10.1186/1471-2261-8-33

**Published:** 2008-11-20

**Authors:** Fernando José Abelha, Susana Quevedo, Henrique Barros

**Affiliations:** 1 Department of Anesthesiology, Hospital de São João, Porto, Portugal; 2 Department of Hygiene and Epidemiology, Hospital de São João, University of Porto Medical School, Porto, Portugal

## Abstract

**Background:**

Most studies documenting beneficial outcomes after carotid endarterectomy (CE) are limited to mortality and morbidity rates, costs, and length of hospital stay (LOS). Few have examined the dependency of patients and how they perceive their own health changes after surgery. The aim of the present study was to evaluate quality of life and independence in activities of daily living (ADL) and to study its determinants.

**Methods:**

Sixty-three patients admitted in the Post Anaesthesia Care Unit (PACU) after CE were eligible for this 14-month follow-up study. Patients were contacted 6 months after discharge to complete a Short Form-36 questionnaire (SF-36) and to have their dependency in ADL evaluated.

**Results:**

Among 59 hospital survivors at 6 months follow-up, 43 completed the questionnaires. Sixty-three percent reported that their general level of health was better on the day they answered the questionnaire than 12 months earlier. Patients had worse SF-36 scores for all domains except bodily pain than a general urban population, and comparison with a group of patients 6 months after surgical ICU discharge showed no differences. Six months after PACU discharge, the Lawton Instrumental Activities of ADL Scale and the Katz Index of ADL demonstrated higher dependency scores (5.9 ± 2.2 versus 4.3 ± 2.4 and 0.3 ± 0.8 versus 0.6 ± 0.9, p < 0.001 and p = 0.047). Sixty-five percent and 33% were dependent in at least one activity in instrumental and personal ADL, respectively. Patients dependent in at least one ADL task had higher Revised Cardiac Risk Index (RCRI) scores (1.0 versus 1.5, p = 0.017). After controlling for multiple comparisons, no significant differences were found.

**Conclusion:**

Patients undergoing CE have improved self-perception of quality of life despite being more dependent. Almost all their scores are worse than those in an urban population. We could identify no predictors of greater dependency in ADL tasks six months after PACU discharge.

## Background

Patients with symptomatic carotid stenosis derive substantial benefit from carotid endarterectomy (CE) and this benefit is thought to be long-lasting [1-3]. CE reduces symptoms and stroke risk in patients with high-grade carotid artery stenosis [4,5]; patients with high grade asymptomatic disease also benefit [6]. Although some studies have documented beneficial outcomes after this procedure, most reports are limited to mortality and morbidity rates, costs and length of hospital stay (LOS) [7,8]. Few studies have examined dependency in Activities of Daily Living (ADL), quality of life, or patients' views of their own health status after CE [9-12]. Indeed, little is known about the extent and impact of these changes on the long-term outcomes for patients.

In this study we review the characteristics of patients undergoing CE, and study quality of life and dependency in ADL 6 months after surgery.

We also consider risk stratification for this particular procedure [13]. Patients were stratified according to cardiac risk factors [14] that are regarded by some authors as predictors of mortality and hospital LOS, but to the best of our knowledge have never been proposed as predictors of health-related quality of life or dependency in ADL.

Several questionnaires have been validated to study Health-Related Quality of Life (HRQOL) [15-19], mostly multi-item scales. Some of these provide a total score as well as subscales giving information on particular aspects such as mobility. The Short-Form General Health Survey (SF-36) was developed during the Medical Outcomes Study to measure generic health concepts that are relevant across age, disease and treatment groups [20]. It is a self-completed questionnaire covering all aspects of HRQOL, it shows good reliability and validity [20,21], and it has been used for various groups of patients including post-discharge Intensive Care Unit (ICU) patients and those subjected to CE [9].

The ability to care for oneself and live independently is considered a measure of functional outcome after hospitalization [22]. Functional status refers to the level of involvement in activities and is often used as a synonym for performance in ADL[23]. ADL appraisal scales consider functional and instrumental activities. The ability of patients to handle these activities has been assessed by generic or disease-specific tests. Katz's ADL Scale [23] and the Lawton Instrumental ADL [24] have been used for critical care survivors.

The aim of the present study was to evaluate quality of life and autonomy in ADL in patients undergoing CE and to identify its determinants.

## Methods

All patients who underwent CE at our institution and were consecutively admitted to the Post Anesthesia Care Unit (PACU) during a period of 14 months beginning in March 2006 were eligible for the study.

All patients had a carotid artery stenosis of ≥ 65%. Their previous neurological evaluations are summarized in Table 1, which summarizes patient characteristics and outcomes. All patients underwent the operation on a single side only and no patient was enrolled twice.

**Table 1:** Patient characteristics and outcomes (n = 63)

**Variable**	
Age in years, median (P25–75)	70 (60–75)
Age group, n (%)	
≥ 65 years	43 (68)
< 65 years	20 (32)
Sex, n (%)	
Male	48 (76)
Female	15 (24)
Body Mass Index in Kg/m^2^, median (P25–P75)	25 (24–28)
General anesthesia/Regional anesthesia, n. (%)	24 (38)/39 (62)
Duration of anesthesia (min.) median (P25–P75)	160 (140–180)
Temperature at admission on PACU, mean ± sd	35.8 ± 0.81
Troponin I at admission, mean ± sd	0.027 ± 0.22
Hypertension, n (%)	60 (95)
Hyperlipidemia, n (%)	53 (84)
Ischemic heart disease, n (%)	41 (65)
Congestive heart disease, n (%)	5 (8)
Cerebrovascular disease, n (%)	34 (54)
Insulin therapy for diabetes, n (%)	3 (5)
Preoperative serum creatinine > 2 mg/dl	1 (2)
Total RCRI, mean ± sd	1.35 ± 0.85
High-risk patients, n (%)	14 (22.2)
Neurological evaluation	
Asymptomatic	13 (21)
Severe bilateral carotid disease	19 (30)
Amaurosis	4 (6)
Transient ischaemic attack	15 (24)
Stroke with full recovery	6 (10)
Stroke with residual deficit	13 (21)
Katz scale, mean ± sd	0.22 ± 0.72
Dependency in I-ADL, n (%)	7 (11)
Lawton I-ADL scale, mean ± sd	6.1 ± 1.9
Dependency in P-ADL, n (%)	14 (22)
SAPS II, median (P25–75)	16 (12–21)
APACHE II, median (P25–75)	7 (6–10)
PACU length of stay (hours), median (P25–75)	21 (16–22)
Hospital length of stay (days), median (P25–75)	5 (4–7)
Mortality in PACU, n (%)	0 (0)
Mortality in hospital, n (%)	3 (4.8)

RCRI, Revised Cardiac Risk Index I-ADL, Instrumental Activities of Daily Living; P-ADL, Personal Activities of Daily Living; SAPS II, Simplified Acute Physiology Score, PACU, Post Anesthesia Care Unit; P25 and P75 are the 25th and 75th percentiles.

The following variables were recorded on admission to the PACU: age, gender, body mass index, preadmission co morbidities (specifically ischemic heart disease, congestive heart failure, cerebrovascular disease, hypertension, renal insufficiency, diabetes and hyperlipidemia) duration of anesthesia, type of anesthesia, core temperature and blood troponin I blood level. The ICU and in-hospital LOS and mortality were recorded for all patients. Simplified Acute Physiology Score II (SAPS II) [25] and Acute Physiology & Chronic Health Evaluation II (APACHE II) [26] were calculated using standard methods.

Adapting a classification scheme developed by Lee and colleagues, we calculated the Revised Cardiac Risk Index (RCRI) score, assigning one point for each of the following risk factors: high-risk surgery, ischemic heart disease, cerebrovascular disease (defined as history of transient ischemic attack or history of cerebrovascular accident), renal insufficiency and diabetes mellitus. Patients were classified as "high-risk" or "low-risk" as described by Boules et al. [13]. "High-risk" patients were defined as those with at least 1 of the following: myocardial infarction (MI) or exacerbation of congestive heart failure (CHF) within 4 weeks before CE; unstable angina; steroid-dependent chronic obstructive pulmonary disease; prior ipsilateral CE, neck dissection or irradiation; high carotid bifurcation, as identified angiographically (at the C2 level or higher) or intraoperatively by the surgeon; and those undergoing combined cardiac-carotid procedures. MI was defined as an event resulting in elevation of cardiac enzymes or electrocardiographic changes. High-risk patients with CHF included those with ejection fraction less than 30% or New York Heart Association class III or IV symptoms requiring hospitalization.

Functional capacity before surgery was evaluated in terms of the patient's ability to handle personal and instrumental ADL within the first 24 hours after PACU admission. All eligible consenting patients were interviewed directly by a trained investigator. When the patient was unable to respond, the questionnaire was completed by a close family member living in the same household as the patient. This evaluation was repeated along with the SF-36 questionnaire six months after PACU discharge.

### Medical Outcome Survey Short-Form 36 (SF-36)

HRQOL was assessed by the SF-36 [21]. The survey contains 36 questions that evaluate eight health domains considered to be important for patient well-being and health status. These domains reflect physical health, mental health, and the impact of health on daily functioning. The eight multiple-item domains encompass physical functioning (ten items), social functioning (two items), role limitations caused by physical problems (four items), role limitations caused by emotional problems (three items), mental health (five items), energy and vitality (four items), pain (two items) and general perception of health (five items). There is one further unscaled item relating to self-reported changes in the respondent's health status during the past year. For each item, scores are coded, summed and transformed to a scale from 0 (worst possible health state measured by the questionnaire) to 100 (best possible health state). Scores can be aggregated to measures representing a physical health summary scale (consisting of physical functioning, physical role, pain and general health) and a mental health summary scale (vitality, social functioning, emotional role and mental health) [15].

The answers to the question about self-reported changes in health status ("compared to one year ago, how you would rate your health in general now?") were dichotomized as: better, about the same or worse than one year ago.

To minimize distress to the next of kin, each patient's records were checked on the hospital information system after 6 months to ascertain whether he or she was still alive. A copy of a formal letter was sent to all known survivors accompanied by a return envelope and a validated Portuguese SF-36 self-report form [27,28]. This version has been validated for the population of the city of Porto from which the subjects of this report were drawn [29]. Scores were compared with normal values for the population.

Values were compared with urban population normal values and with those collected during another study in a general surgical population admitted to an ICU located in the same demographic area [30].

### Activities of Daily Living (ADL)

The questionnaire used to assess dependency was based on the Katz Index of Independence in ADL [22] and Lawton Instrumental ADL scale. The Lawton IADL scale is an easily to administer assessment instrument that provides self-reported information about the functional skills necessary for living in the community. Deficits in the instrumental Lawton scale were scored and a summary score ranging from 0 (low function, dependent) to 7 (high function, independent) was obtained. The Katz ADL scale assesses basic personal activities of daily living and ranks adequacy of performance in six functions. Dependency in each personal activity was evaluated and a summary score ranging from 0 (independence in all activities) to 6 (dependency in all activities) was obtained. The personal ADL (P-ADL) considered were bathing, dressing, going to the toilet, transferring from bed to chair, continence and feeding. The instrumental ADL (I-ADL) considered were cleaning, food shopping, public transportation and cooking. Answers were categorized into two groups: able or unable to perform each activity and group of activities. Four categories were possible: (a) I-ADL and P-ADL independent, (b) I-ADL dependent but P-ADL independent, (c) P-ADL dependent but I-ADL independent and (d) both P-ADL and I-ADL dependent. Patients were considered dependent if they were dependent in at least one I-ADL or P-ADL activity.

The study protocol was approved by our institutional review board and written consent was obtained from all patients or members of their families.

### Statistical methods

Descriptive analyses of variables were used to summarize data and the Mann-Whitney U test was used to compare continuous variables between two groups of subjects; chi-square or Fischer's exact test were used to compare proportions between two groups of subjects.

We used a significance level of 0.05 (two sided) for almost all statistical tests except when multiple comparisons were made. In such cases we controlled the values for multiple comparisons to reduce the risk of type II error. SPSS for Windows version 13.0 (SPSS, Chicago, IL) was used to analyze the data.

A t test for independent groups was used for comparison to population means.

The CE population and the general population were compared using a paired t test. Every patient in the CE population was paired prospectively with a demographically matched patient from the control population.

## Results

During the study period, 63 patients were admitted to the PACU. The mean patient age was 70 years (range 44–84), with most CEs performed on males (76%). Median SAPS II was 16 (range 7–65), median APACHE II was 7 (range 2–22) and median LOS in hospital was 5 days. Patient characteristics and outcomes are summarized in Table 1.

There were no perioperative deaths, and morbidity was limited to 1 acute myocardial infarction and 3 thrombotic strokes.

Three patients died in hospital and one died before the 6-month evaluation (6.3% mortality rate at the time of evaluation). Of the remaining 59 patients, 14 (24%) did not answer the questionnaires at 6 months follow-up but were known to be alive. The characteristics and outcomes of patients who completed the study are presented in Table 2.

**Table 2:** Comparison of characteristics and outcomes between respondents and non-respondents

**Variable**	**Respondents** **(n = 43)**	**Non-respondents** **(n = 16)**	**p value**
Age in years, median (P25–75)	68 (60–75)	71 (59–77)	0.585^b^
Sex, n (%)			0.589^a^
Male	33 (77)	12 (75)	
Female	10(23)	4 (25)	
BMI (Kg/m^2^), median (P25–P75)	26 (24–28)	25 (24–29)	0.809^b^
Duration of anesthesia (min.) median (P25–P75)	150 (120–180)	178 (150–206)	0.286^b^
General anesthesia/Regional anesthesia	16/27	6/10	0.606^a^
Total RCRI, median (P25–P75)	1 (1–2)	2 (1–2)	0.173^b^
Temperature at admission	35.77 ± 0.85	36.08 ± 0.67	0.276^b^
Troponin I at admission	0.02 ± 0.02	0.03 ± 0.03	0.278^b^
High-risk patients, n (%)	8 (19)	5 (31)	0.241^a^
Hypertension, n (%)	41 (95)	15 (94)	0.620^a^
Ischemic heart disease, n (%)	28 (65)	10(63)	0.595^a^
Congestive heart disease, n (%)	3 (7)	1(6)	0.705^a^
Cerebrovascular disease, n (%)	19 (44)	13(81)	0.011^a^
Insulin therapy for diabetes, n (%)	1 (2)	1(6)	0.472^a^
Preoperative serum creatinine > 2 mg/dl, n (%)	1(2)	0 (0)	0.729^a^
Hyperlipidemia, n (%)	36(84)	13(81)	0.549^a^
Dependency in I-ADL, n (%)	5 (12)	1(6)	0.477^a^
Dependency in P-ADL, n (%)	11(26)	3(19)	0.431^a^
Previous Katz, mean (SD)	0.26 ± 0.82	0.13 ± 0.5	0.548^b^
Previous Lawton, mean (SD)	5.91 ± 2.20	6.50 ± 1.16	0.493^b^
SAPS II, median (P25–75)	17 (12 – 20)	16 (12 – 22)	0.986^b^
APACHE II, median (P25–P75)	7 (6–9)	8 (6–11)	0.945^b^
Hours of ICU length of stay, median (P25–75)	20 (16–22)	20 (16–22)	0.797^b^
Days of hospital length of stay, median (P25–P75)	5 (4–7)	6 (5–8)	0.150^b^

^a ^Pearson χ^2^. ^b ^Mann-Whitney test.

There were no significant differences after controlling for multiple comparisons

BMI, Body Mass Index; RCRI, Revised Cardiac Risk Index; I-ADL, Instrumental Activities of Daily Living; P-ADL, Personal Activities of Daily Living; SAPS II, Simplified Acute Physiology Score, PACU, Post Anesthesia Care Unit; P25 and P75 are the 25^th ^and 75^th ^percentiles.

Participating patients (response rate 76%) more frequently had histories of cerebrovascular disease (defined by Lee et al. for RCRI [14] as history of transient ischemic attack or of cerebrovascular accident) and there were no statistical significant differences between participants and non-participants regarding the other variables studied (Table 2).

### Functional capacity and ADL

Six months after discharge from ICU, 65% of the patients were dependent in at least one activity in instrumental ADL and 33% in at least one personal ADL (Table 3).

**Table 3:** Dependency and self-reported changes in health in general, 6 months after PACU discharge (n = 38)

**Variable**	**Before surgery**	**6 months after EC**	**P**
ADL			
Personal			
Katz scale	0.26 ± 0.82	0.56 ± 0.94	**0.047**
Dependency in P-ADL, n (%)	5 (12)	14 (33)	0.186
Instrumental			
Lawton scale	5.91 ± 2.20	4.28 2.43	**< 0.001**
Dependency in I-ADL, n (%)	11 (26)	28 (65)	0.164

I-ADL, Instrumental Activities of Daily Living; P-ADL, Personal Activities of Daily Living

Dependency in P-ADL was significantly more frequent after surgery and scores on the Katz and Lawton scales were significantly different, indicating more dependency in ADL after surgery.

The only variable with p < 0.05 on the Pearson χ^2 ^and Mann-Whitney tests was the RCRI score. This result was no longer significant after application of Bonferroni's correction for multiple comparisons (Table 4).

**Table 4:** Comparisons for patients with dependency in ADL 6 months after EC

	**ADL Dependency**		
**Variable**	**No (n = 15)**	**Yes (n = 28)**	**P**
Age, median	67	70	0.365^b^
Gender, n(%)			0.231^a^
Female	2 (13)	8 (29)	
Male	13 (87)	20 (71)	
BMI, median	25.7	25.7	0.908^b^
Duration of anesthesia (min.), median	150	180	0.057^a^
T ype of anesthesia, n(%)			0.239^a^
General	4 (27)	12 (43)	
Local	11 (73)	16 (57)	
High-risk patients, n (%)	2 (13)	6 (21)	0.417^a^
Temperature at admission	35.6	36.0	0.474^b^
Troponin I at admission	0.001	0.002	0.178^b^
Hypertension, n(%)	14 (93)	27 (96)	0.581^a^
Hyperlipidemia, n(%)	12 (80)	24 (86)	0.468^a^
Ischemic heart disease, n(%)	7 (47)	21 (75)	0.065^a^
Congestive heart disease	0	3 (11)	0.265^a^
Cerebrovascular disease	5 (33)	14 (50)	0.235^a^
Insulin therapy for diabetes	1 (7)	0	0.349^a^
Preoperative serum creatinine > 2 mg/dl	0	1 (4)	0.651^a^
RCRI score, median	1.0	1.5	0.017^b^
SAPS II, median	15.0	17.0	0.239^b^
APACHE II, median	7.0	7.0	0.301^b^
Length of PACU stay (hours), median	19.0	20.0	0.768^b^
Length of Hospital stay (days), median	5.0	5.0	0.326^b^

^a ^Pearson χ^2^. ^b ^Mann-Whitney test.

There were no significant differences after controlling for multiple comparisons

BMI, Body mass index; RCRI, Revised Cardiac Risk Index; SAPS II, Simplified Acute Physiology Score; APACHE II, Acute Physiology and Chronic Health Evaluation; PACU, Post Anesthesia Care Unit;

#### Quality of Life Measures

Overall, 63% stated that their level of health in general was better on the day they completed the SF-36 while 11% considered it to be worse than previously (6 months before PACU discharge). There was no statistically significant relationship between the patients' baseline characteristics and a worse self-reported general level of health.

Compared to normal values for the urban population of Porto, the SF-36 subscores of all patients were worse on all domains except bodily pain (Table 5). Compared to the mean values for non-cardiac surgical patients from the same urban area 6 months after ICU discharge [30], no differences were observed on any domain (Table 6).

**Table 5:** SF-36 after CE and in a general population

**Variable**	**6 months after CE**	**General population**	**p**
SF-36 domains, mean ± sd			
Physical function	52.0 ± 25.9	75.4 ± 23.6	< 0.001^a^
Role physical	50.3 ± 34.7	76.7 ± 26.1	< 0.001^a^
Bodily pain	60.9 ± 27.8	65.7 ± 26.2	0.11^a^
General health perception	46.8 ± 25.7	59.5 ± 19.8	< 0.001^a^
Vitality	35.0 ± 14.9	57.2 ± 21.1	< 0.001^a^
Social functioning	57.6 ± 26.9	76.0 ± 24.1	< 0.001^a^
Role emotional	53.1 ± 34.3	76.9 ± 25.8	< 0.001^a^
Mental health	50.9 ± 17.2	66.1 ± 22.8	< 0.001^a^

^a ^paired t test

SF-36, Short-form 36; CE, carotid endarterectomy

Sd, standart deviation

**Table 6:** SF-36 after CE and in a surgical population

**Variable**	**6 months after CE**	**ICU surgical patients**	**p**
SF-36 domains, mean ± sd			
Physical function	52.0 ± 25.9	54.1 ± 29.0	0.329^a^
Role physical	50.3 ± 34.7	45.2 ± 33.8	0.183^a^
Bodily pain	60.9 ± 27.8	57.7 ± 29.8	0.258^a^
General health perception	46.8 ± 25.7	44.5 ± 26.6	0.301^a^
Vitality	35.0 ± 14.9	34.7 ± 17.0	0.457^a^
Social functioning	57.6 ± 26.9	60.2 ± 28.7	0.291^a^
Role emotional	53.1 ± 34.3	50.1 ± 34.2	0.299^a^
Mental health	50.9 ± 17.2	48.6 ± 22.3	0.261^a^

^a ^paired t test

SF-36, Short-form 36; CE, carotid endarterectomy

Sd, standart deviation

## Discussion

Carotid endarterectomy is performed to prevent stroke and its complications, namely death or a decrease in quality of life [4,31]. Evidence supports that this procedure is the standard treatment of severe carotid stenosis being a safe and effective procedure in the general population. In a recent study of Ballotta et al. among 348 patients with a complete follow-up after CE, the 5- and 10-year risk of death was 3.7% and 14.3%, respectively [32].

Vriens et al. reported that CE does not disrupt quality of life [33]. The assessment of quality of life represents an important means of examining how disability or cognitive impairment impacts upon a patient's day-to-day life. The combination of cognitive function and quality of life data could provide a more global impression of the impact of the surgery on patients. The effect of CE on cognitive function is controversial [34]. Many studies demonstrate subtle cognitive changes as revealed by neuropsychological testing. Several studies have demonstrated improvement [35,36] whereas others show no change[37,38], and still others demonstrate a decline [39,40] in postoperative neuropsychological performance. Diversity of the patient population, variability of the surgical technique, differences in neuropsychological tests, and varying follow-up periods are all factors contributing to the difficulty in obtaining consistent results among studies. Adding to the complexity, many groups report significant decline in some cognitive domains and significant improvements in others [39].

With the present study we have examined the impact of CE on quality of life and independence in activities of daily living. To study the impact of the procedure in quality of life we have used the self evaluated health transition item of SF-36 questionnaire. This item is not used in scoring the scales and has been shown to be useful in estimating average change in health status during the year prior to its administration [41]. Measuring changes in health status and as reported by Dardik A [12] we found an improved subjective perception of quality of life after CE among the patients who completed the study. Other studies on patients after ICU discharge have reported similar findings using different tools [30,42]. Because self-perception of health may reflect anticipation of future health after a surgical procedure, it is not surprising that these patients, who view themselves as chronically ill, have scores identical to those of other chronically ill patients subjected to surgical procedures and admitted to an ICU (27). Comparisons with a general (taken as "control") population are difficult to interpret because patients subjected to CE generally perceive themselves as chronically ill. Thus, our finding that quality of life was worse in our patients than in the general population was not totally unexpected. ICU patients with similar demographic characteristics from the same urban area seemed more appropriate for establishing comparisons with our patients. This was done and the results were similar to those of Dardik et al. [12], who compared the SF-36 subscores of 50 patients against the population normal values for both healthy and chronically ill subjects in the same age range. They found that the mean postoperative physical function subscores were similar to those of the chronically ill population, whereas the emotional subscores were similar to the mean values of the healthy population.

The patients in our study had higher degrees of dependency in instrumental and personal ADL after surgery, which was not entirely unexpected in view of the extent of comorbidities and the natural history of their atherosclerotic disease. It seems paradoxical that these patients, despite being more dependent, stated that their quality of life was better than before surgery. We think this could also be explained by their expectation of better health when they agreed to surgical intervention.

Trudel et al. concluded that quality of life seemed more markedly affected by cardiovascular and neurological problems. In our study, total RCRI was the only determinant of dependency six months after EC. We did not consider this to be strong enough for true statistical significance because the difference was not consistent after the data were controlled for multiple comparisons.

Trudel and coworkers [43] found that only 20% of patients after CE met the criteria for normal functional capacity. These results are somewhat worse than our findings, although the methods used to classify dependency were different and the study was written more than 20 years ago when surgical skills and procedures for CE were less developed.

The overall characteristics of non-respondents were similar to those of participants and the only observed difference was the incidence of cerebrovascular disease, which was higher in the participants. It must be emphasized that poor quality of life or high incidence of psychological disturbance at the time of the follow-up survey may have contributed to non-response to the questionnaire, constituting a possible limitation of this study [44,45].

This study has several limitations. The small sample size may limit the ability to document real differences among our subgroups of patients. We did not apply the SF-36 questionnaire before surgery so it was not possible to compare quality of life of patients before and after surgery as in the study by Lloyd [10]. Nevertheless, we used the SF-36 question about self-reported changes in health status ("compared to one year ago, how would you rate your health in general now?") to conclude that the level of health in general was better for most patients on the day they completed the SF-36 than before surgery.

Table 4 shows p values < 0.05 in the statistical tests for differences in ADL independence, but when these data where controlled for multiple comparisons to reduce the risk that they were due to chance, they showed no statistical significance. So these results were considered not strong enough to infer a real difference.

## Conclusion

In summary, this study supports the conclusion that patients subjected to carotid endarterectomy perceive their quality of life as improved six months after surgery although they are more dependent in ADL activities.

## Competing interests

The authors declare that they have no competing interests.

## Authors' contributions

All people listed as authors contributed to the preparation of the manuscript and no person or persons other than the authors listed have contributed significantly to its preparation.

Each listed author participated in the work to the extent that they could all publicly defend its content. They all read the manuscript before its submission for publication and are prepared to sign a statement stating they had read the manuscript and agree to its publication.

## Pre-publication history

The pre-publication history for this paper can be accessed here:


